# Internal herniation of the cecum through the foramen of Winslow—a case report

**DOI:** 10.1093/jscr/rjab459

**Published:** 2021-10-31

**Authors:** Emmanuel Luciano, Russell Hyde, Wael Solh, Ryan T Davis, Felipe Pacheco

**Affiliations:** Central Michigan University College of Medicine, Saginaw MI, USA; Central Michigan University College of Medicine, Saginaw MI, USA; Central Michigan University College of Medicine, Saginaw MI, USA; Central Michigan University College of Medicine, Saginaw MI, USA; Central Michigan University College of Medicine, Saginaw MI, USA

## Abstract

Foramen of Winslow hernias are a rare, but dangerous form of internal hernia that can present in individuals with signs and symptoms of bowel obstruction. This case report details operative management of a cecal herniation through the foramen of Winslow in an elderly male with no prior history of intra-abdominal surgery. The patient presented with worsening abdominal pain, nausea, vomiting and obstipation. Due to the clinical picture of a complete bowel obstruction and subsequent imaging findings, an urgent abdominal exploration was performed. During the procedure, the cecum was found to be ischemic and strangulated in the lesser sac, herniated through the foramen of Winslow. Following operative reduction and right hemicolectomy, it was decided to close the foramen of Winslow to prevent recurrence and future complications. The patient had an uncomplicated postoperative course with resolution of symptoms.

## INTRODUCTION

Internal hernias through the foramen of Winslow, also known as Blandin’s hernia, account for 8% of internal hernias and 0.08% of all hernias [1–3]. Initially described by Blandin in 1834, <10% of cases are diagnosed preoperatively leading to high mortality rates when care is delayed reaching up to 50% [4, 5]. This mortality rate has been reduced to ~5% with early surgical intervention and use of computed tomography (CT; [3, 4]). Even with widespread use of cross-sectional imaging, this type of internal hernia remains extremely difficult to diagnose with potential life-threatening complications, such as strangulated bowel [4, 6]. The vast majority of these cases are managed with laparotomy with only a few case reports favoring a laparoscopic approach [3–5]. This type of hernia most commonly presents in middle-aged adults with signs of intestinal obstruction and a surgical abdomen [7, 8]. Herniation of the small bowel accounts for 63%, whereas cecal herniations account for <5% of foramen of Winslow hernias [7]. This case report details the operative management of a cecal herniation through the foramen of Winslow.

## CASE REPORT

**Figure 1:**
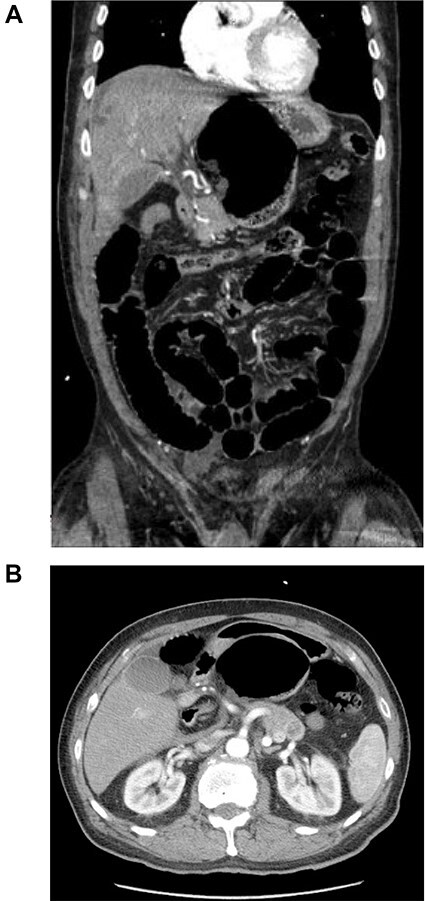
CT scan of the abdomen and pelvis with IV contrast. (**A**) Coronal view shows internal herniation of the cecum through the foramen of Winslow. (**B**) Axial view demonstrates the same findings with the stomach displaced both laterally and anteriorly by the cecum. Of note there was 1.5-cm ring-enhancing lesion located in the body of the pancreas found incidentally.

**Figure 2:**
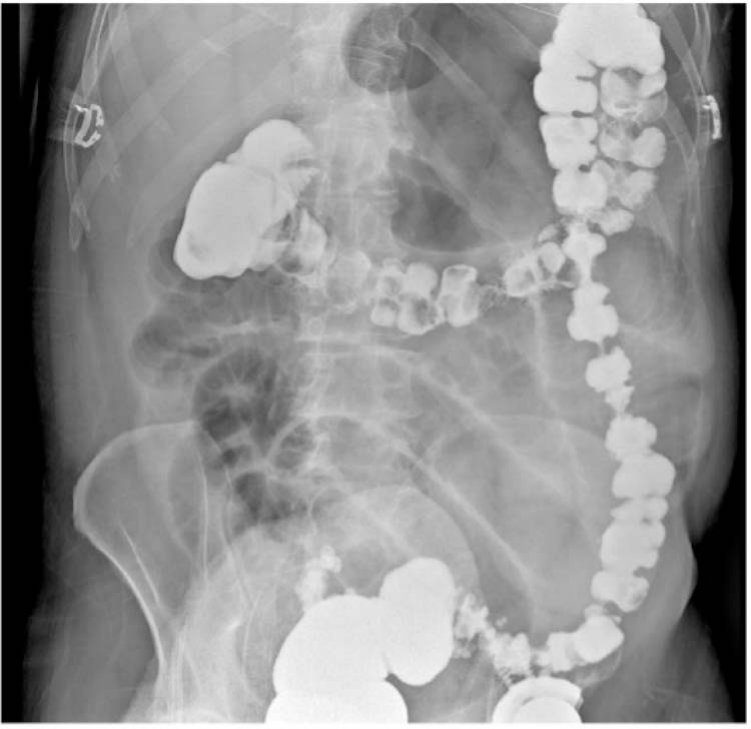
Barium enema demonstrating a transition point in the hepatic flexure with contrast angling towards the lesser sac.

A 67-year-old male with a past medical history of prostate cancer, hypertension and prior open right inguinal hernia repair presented to the emergency department (ED) with sudden onset nausea, vomiting and abdominal pain for 2 h. The abdomen was non-distended and slightly tender in the epigastrium. A clinical diagnosis of pancreatitis was made based on physical examination, history and a mildly elevated lipase and the patient was discharged home with antiemetic medications by the ED providers. The patient presented back to the ED 2 days later with worsening abdominal pain, nausea, vomiting and obstipation. At this time his abdomen was distended and tender to palpation in the epigastrium without peritoneal irritation. Due to his obstructive clinical picture, a CT scan of the abdomen and pelvis with IV contrast (Fig. 1) was obtained in the ED. The scan was read as a possible volvulus versus an internal hernia causing small bowel obstruction. A nasogastric tube was placed after he was admitted to the surgical unit with partial resolution of his abdominal tenderness and distention. A barium enema (Fig. 2) was performed to rule out an obstructing distal mass, which showed a transition point at the hepatic flexure.

Due to the clinical picture of a complete bowel obstruction and subsequent imaging findings, an urgent abdominal exploration was performed. A standard supraumbilical midline laparotomy was made and the peritoneal cavity was entered. Distended loops of bowel were encountered, and the right colon was mobilized from some loose attachments to the abdominal wall. The right colon was followed to identify the cecum and terminal ileum. The cecum was identified to be in the lesser sac, herniated through the foramen of Winslow. The cecum was reduced and was determined to be ischemic and nonviable, so a right hemicolectomy was performed with an isoperistaltic ileocolic anastomosis with the transverse colon. The duodenum was Kocherized to expose the retroperitoneum. The foramen of Winslow was closed by approximating the hepatoduodenal ligament to peritoneum overlying the right kidney with a simple interrupted 3–0 polyglyconate suture, adequately obliterating the foramen. The transverse colon was pexy to the anterior abdominal wall using 3–0 polyglyconate suture.

His postoperative course was uncomplicated. His intestinal transit was restored on postoperative Day 3. His diet was advanced as tolerated. He was discharged on Day 5 after surgery with good diet tolerance and having adequate bowel function. Pathology was benign.

## DISCUSSION

Diagnosing a foramen of Winslow hernia preoperatively remains challenging with only 10% of cases diagnosed preoperatively [9]. Specifically, in this case, both the rarity of disease and complex radiographic findings proved to be barriers for early diagnosis. The foramen of Winslow hernia being detected retrospectively is a common occurrence amongst surgical teams handling these cases [9]. The decision to close the foramen was to prevent recurrence and future complications; however, some cases report enlarging the foramen of Winslow to prevent future strangulation, while others have decided to close the defect with omentum with similar success [7].
